# Development of PD-1 and PD-L1 inhibitors as a form of cancer immunotherapy: a comprehensive review of registration trials and future considerations

**DOI:** 10.1186/s40425-018-0316-z

**Published:** 2018-01-23

**Authors:** Jun Gong, Alexander Chehrazi-Raffle, Srikanth Reddi, Ravi Salgia

**Affiliations:** 1 Department of Medical Oncology, City of Hope National Medical Center, 1500 E Duarte St, Duarte, CA 91010 USA; 2 Department of Internal Medicine, Harbor-UCLA Medical Center, 1000 W Carson St, Torrance, CA 90509 USA; 3 Medical Oncology and Experimental Therapeutics, City of Hope Comprehensive Cancer Center, Building 51, Room 101, 1500 E Duarte St, Duarte, CA 91010 USA

**Keywords:** PD-1 inhibitor, PD-L1 inhibitor, Clinical trials, Biomarkers, Immune checkpoint, Hyperprogressors, Treatment beyond progression, Microbiome, Immune-related toxicity

## Abstract

Early preclinical evidence provided the rationale for programmed cell death 1 (PD-1) and programmed death ligand 1 (PD-L1) blockade as a potential form of cancer immunotherapy given that activation of the PD-1/PD-L1 axis putatively served as a mechanism for tumor evasion of host tumor antigen-specific T-cell immunity. Early-phase studies investigating several humanized monoclonal IgG4 antibodies targeting PD-1 and PD-L1 in advanced solid tumors paved way for the development of the first PD-1 inhibitors, nivolumab and pembrolizumab, approved by the Food and Drug Administration (FDA) in 2014. The number of FDA-approved agents of this class is rapidly enlarging with indications for treatment spanning across a spectrum of malignancies. The purpose of this review is to highlight the clinical development of PD-1 and PD-L1 inhibitors in cancer therapy to date. In particular, we focus on detailing the registration trials that have led to FDA-approved indications of anti-PD-1 and anti-PD-L1 therapies in cancer. As the number of PD-1/PD-L1 inhibitors continues to grow, predictive biomarkers, mechanisms of resistance, hyperprogressors, treatment duration and treatment beyond progression, immune-related toxicities, and clinical trial design are key concepts in need of further consideration to optimize the anticancer potential of this class of immunotherapy.

## Background

**Fig. 1:**
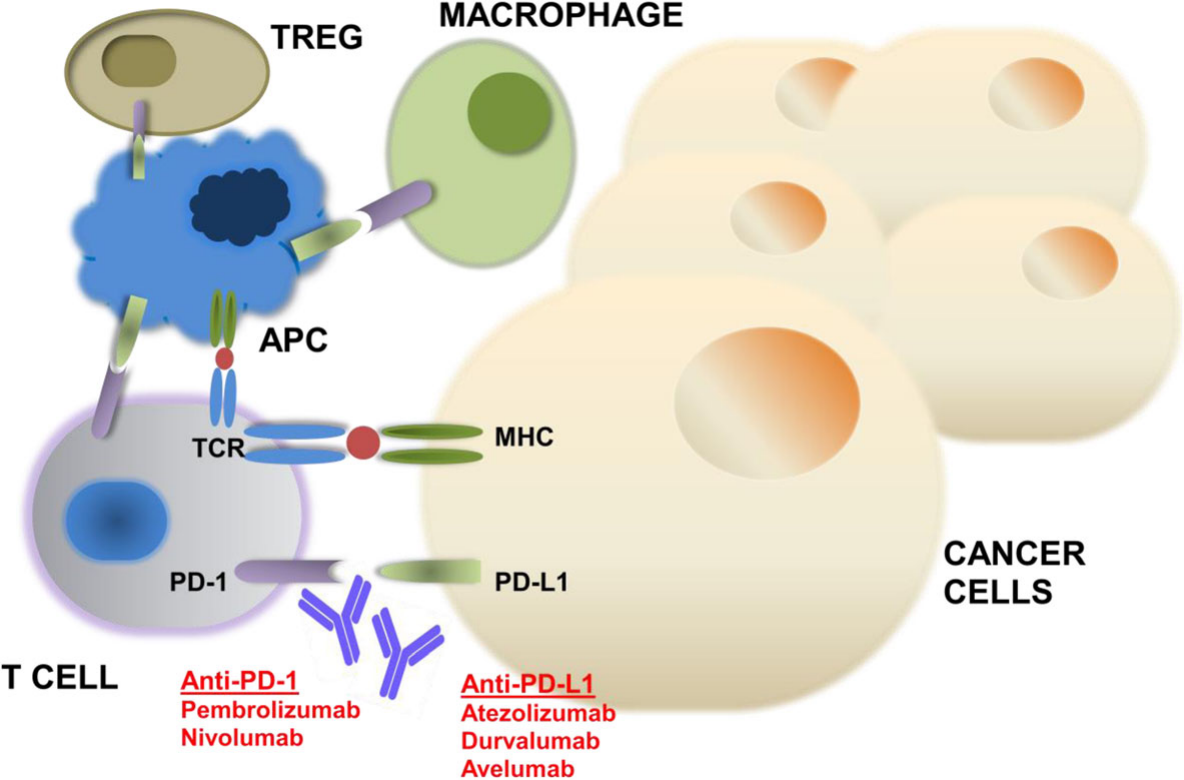
Mechanism of action of PD-1 and PD-L1 inhibitors. The programmed cell death 1 (PD-1) receptor is expressed on activated T cells, B cells, macrophages, regulatory T cells (Tregs), and natural killer (NK) cells. Binding of PD-1 to its B7 family of ligands, programmed death ligand 1 (PD-L1 or B7-H1) or PD-L2 (B7-DC) results in suppression of proliferation and immune response of T cells. Activation of PD-1/PD-L1 signaling serves as a principal mechanism by which tumors evade antigen-specific T-cell immunologic responses. Antibody blockade of PD-1 or PD-L1 reverses this process and enhances antitumor immune activity. TCR, T-cell receptor; MHC, major histocompatibility complex; APC, antigen-presenting cell

The programmed cell death protein 1 receptor (PD-1) receptor was first described in the early 1990s given its expression during induction of apoptosis in a T-cell hybridoma [1, 2]. Since its initial discovery several groups have identified that engagement of PD-1 through its ligand, programmed death ligand 1 (PD-L1), negatively regulates T-cell-mediated immune responses [3–6]. Early preclinical evidence suggested that activation of PD-1/PD-L1 signaling could serve as a mechanism for tumors to evade an antigen-specific T-cell immunologic response [6–8]. Consequently, the hypothesis was developed that PD-1/PD-L1 blockade may be an effective cancer immunotherapy (Fig. 1).

Initial phase I studies investigating several humanized monoclonal IgG4 antibodies targeting PD-1 and PD-L1 in advanced solid tumors were soon conducted and paved way for the development of the first PD-1 inhibitors, nivolumab and pembrolizumab, approved by the Food and Drug Administration (FDA) [9–11]. Immune checkpoint inhibitors targeting the PD-1/PD-L1 axis are now approved in the treatment of several malignancies ranging from classical Hodgkin lymphoma to head and neck squamous cell carcinoma (HNSCC) [12].

**Fig. 2:**
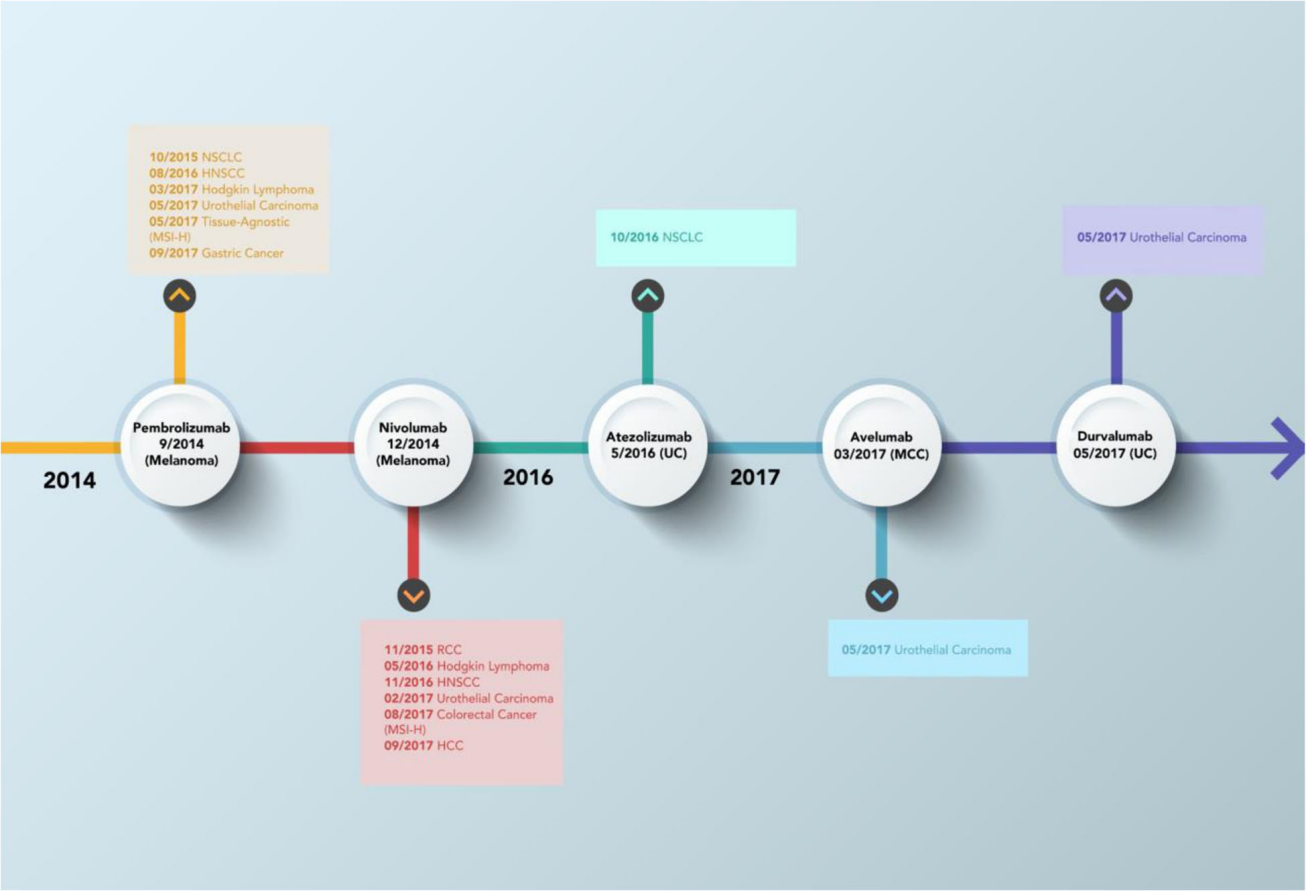
Timeline of FDA approvals for PD-1 and PD-L1 inhibitors in cancer therapy. The Food and Drug Administration approvals of programmed cell death 1 (PD-1) and programmed death ligand 1 (PD-L1) inhibitors detailed by agent, date of approval, and tumor type. NSCLC, non-small cell lung cancer; HNSCC, head and neck squamous cell carcinoma; MSI-H, microsatellite instability-high; RCC, renal cell carcinoma; HCC, hepatocellular carcinoma; UC, urothelial carcinoma; MCC, Merkel cell carcinoma

Since the approval of pembrolizumab for the treatment of advanced melanoma in September 2014, the clinical development of PD-1 and PD-L1 inhibitors as anticancer agents has broadened (Table 1). Presently, the FDA has approved PD-1/PD-L1 inhibitors for the treatment of nine cancer types (Fig. 2). The purpose of this review is to highlight the clinical development of PD-1 and PD-L1 inhibitors in cancer therapy to date. In particular, we focus on detailing the registration trials that have led to FDA-approved indications of anti-PD-1 and anti-PD-L1 therapies in cancer and discuss future considerations important to optimizing their antitumor efficacy.

**Table 1:** Overview of PD-1/PD-L1 inhibitors, mechanisms of action, trial designations and approved companion diagnostics

Agent	Mechanism of action	Trial name(s)	FDA-approved PD-L1 companion diagnostic
Pembrolizumab	PD-1 inhibitor	KEYNOTE	Primary antibody: 22C3 (Dako) IHC scoring: Tumor cell membrane Therapeutic developer: Merck
Nivolumab	PD-1 inhibitor	CheckMate	Primary antibody: 28-8 (Dako) IHC scoring: Tumor cell membrane Therapeutic developer: BMS
Atezolizumab	PD-L1 inhibitor	IMVigor (UC), POPLAR (NSCLC), OAK (NSCLC)	Primary antibody: SP142 (Ventana) IHC scoring: Tumor cell membrane, infiltrating immune cells Therapeutic developer: Genentech
Durvalumab	PD-L1 inhibitor	Study 1108	Primary antibody: SP263 (Ventana) IHC scoring: Tumor cell membrane Therapeutic developer: AstraZeneca
Avelumab	PD-L1 inhibitor	JAVELIN	Primary antibody: 73-10 (Dako)^a^ IHC scoring: Tumor cell membrane Therapeutic developer: Merck, Pfizer

*PD-1* programmed cell death 1, *PD-L1* programmed death ligand 1, *FDA* Food and Drug Administration, *IHC* immunohistochemistry, *BMS* Bristol-Myers Squibb, *UC* urothelial carcinoma, *NSCLC* non-small cell lung cancer

^a^ For research use only

A literature search was conducted in MEDLINE using the following key words: “programmed death 1,” programmed death-ligand 1,” “PD-1,” “PD-L1,” “immune checkpoint inhibitor” and limited to published studies of English language up to October 1, 2017. Studies were further restricted to registration trials leading to FDA-approved indications in cancer therapy. An additional manual search was performed to include preliminary results from abstracts of potential relevance.

## Melanoma

### Pembrolizumab

On September 4, 2014, pembrolizumab (humanized monoclonal IgG4 antibody) became the first PD-1 inhibitor to receive approval for patients with advanced or unresectable melanoma based on the findings from the KEYNOTE-001 study [13, 14]. In this phase I multicenter, international, open-label, randomized expansion of the KEYNOTE-001 cohort, 173 patients with advanced or unresectable melanoma who had previously failed treatment with ipilimumab and a BRAF inhibitor (if *BRAF*^*V600*^-mutated) were treated with pembrolizumab [14]. Patients were randomly assigned to treatment with pembrolizumab intravenous (IV) at 2 mg/kg every 3 weeks or 10 mg/kg every 3 weeks. The primary study endpoint was overall response rate (ORR) per RECIST 1.1. The ORR was 26% in both the pembrolizumab 2 mg/kg and 10 mg/kg groups (Table 2). Grade 3-4 drug-related adverse events (AEs) occurred in 15% of the pembrolizumab 2 mg/kg group (most common fatigue 6%) and 8% of the pembrolizumab 10 mg/kg group (1 each of diarrhea, rash, dyspnea, hypoxia, maculopapular rash, pancreatitis, and musculoskeletal pain) [14]. In an update of KEYNOTE-001, findings after a median follow-up duration of 18 months for all patients were published [15]. Progression-free survival (PFS) at 6 months was 45%, median overall survival (OS) was 25.9 months, and ORR 34% in ipilimumab-treated and 45% in ipilimumab-naïve patients. Pembrolizumab was well tolerated as 14% of all patients experienced grade ≥ 3 AEs.

**Table 2:** Registration trials leading to the FDA approval of PD-1/PD-L1 inhibitors in melanoma

Study/Agent	Tumor (*n*)	Line of therapy	Experimental arm	Control arm	Primary endpoint^a^	Ref
KEYNOTE-001 (phase I)/pembrolizumab	Advanced melanoma (*n* = 173)	Previously treated with ipilimumab and/or BRAF inhibitor	Pembrolizumab 2 mg/kg or 10 mg/kg every 3 weeks		ORR 26% (both doses; difference 0%, 95% CI 14-13, *p* = 0.96)	14
KEYNOTE-006 (phase III)/pembrolizumab	Advanced melanoma (*n* = 834)	First-line (regardless of *BRAF* mutations status)	Pembrolizumab 10 mg/kg every 2 weeks OR every 3 weeks	Ipilimumab 3 mg/kg every 3 weeks X4 cycles	PFS (6-month) 47.3% vs. 46.4% vs. 26.5% (HR 0.58 for both pembrolizumab regimens vs. ipilimumab 95% CI 0.46-0.72 and 0.47-0.72, respectively, *p* < 0.001) OS (1-year) 74.1% vs. 68.4% vs. 58.2% (HR pembrolizumab every 2 weeks 0.63, 95% CI 0.47-0.83, *p* = 0.0005; HR pembrolizumab every 3 weeks 0.69, 95% CI 0.52-0.90, *p* = 0.0036)	16
KEYNOTE-002 (phase II)/pembrolizumab	Advanced melanoma (*n* = 540)	Refractory to ipilimumab and/or BRAF inhibitor	Pembrolizumab 2 mg/kg every 3 weeks OR 10 mg/kg every 3 weeks	ICC (paclitaxel+carboplatin, paclitaxel, carboplatin, dacarbazine, or temozolomide)	PFS 2 mg/kg (HR 0.57 95% CI 0.45-0.73, p < 0.001) and 10 mg/kg (HR 0.50, 95% CI 0.39-0.64, *p* < 0.001) compared to ipilimumab No superiority in OS at this interim analysis	17
CheckMate 037 (phase III)/nivolumab	Stage IIIC or IV melanoma (*n* = 405)	Second-line	Nivolumab 3 mg/kg every 2 weeks	Dacarbazine 1000 mg/m2 every 3 weeks OR carboplatin AUC 6 + paclitaxel 175 mg/m^2^ every 3 weeks	ORR 31.7% (95% CI 23.5-40.8) vs. 10.6% (95% 3.5-23.1)	18
CheckMate 069 phase III)/nivolumab/ipilimumab	*BRAF*^*V600*^-WT unresectable or metastatic melanoma (*n* = 142)	First-line	Nivolumab 1 mg/kg + ipilimumab 3 mg/kg every 3 weeks X4 cycles then nivolumab alone every 2 weeks	Ipilimumab 3 mg/kg every 3 weeks	ORR 61% vs. 11% (p < 0.001)	19
CheckMate 067 phase III)/nivolumab/ipilimumab	Unresectable or metastatic melanoma (*n* = 945)	First-line	Arm 1: Nivolumab 3 mg/kg every 2 weeks Arm 2: nivolumab 1 mg/kg and ipilimumab 3 mg/kg every 3 weeks for 4 doses followed by nivolumab 3 mg/kg of every 2 weeks	Ipilimumab 3 mg/kg every 3 weeks	PFS 6.9 mos (HR compared to ipilimumab 0.57, 99.5% CI 0.43-0.76, p < 0.001 vs. 11.5 mo (HR 0.42, 99.5% CI 0.31-0.57, *p* < 0.001 compared to ipilimumab) vs. 2.9 mos	20

^a^ Order of results refers to the experimental arm and control arm, respectively. In trials with more than one experimental arm, the endpoints are in the same order as documented in the experimental arm column

*FDA* Food and Drug Administration, *PD-1* programmed cell death 1, *PD-L1* programmed death ligand 1, *ORR* overall response rate, *CI* confidence interval, *PFS* progression-free survival, *HR* hazard ratio, *OS* overall survival, *ICC* investigator-choice chemotherapy, *AUC* area under curve, *WT* wild-type

On December 18, 2015, pembrolizumab received an expanded first-line indication to include previously-untreated advanced melanoma regardless of *BRAF* mutation status following the results of the KEYNOTE-006 trial [16]. In this international, randomized, open-label phase 3 study, pembrolizumab 10 mg/kg every 2 weeks or every 3 weeks vs. ipilimumab 3 mg/kg every 3 weeks was evaluated in patients with advanced, unresectable stage III or IV melanoma who had received ≤1 previous systemic therapy for advanced disease. Primary endpoints were PFS and OS and 6-month PFS for patients who received pembrolizumab every 2 weeks and every 3 weeks was 47.3% and 46.4%, respectively, compared to 26.5% for those who received ipilimumab (hazard ratio (HR) for disease progression 0.58 for both pembrolizumab regimens vs. ipilimumab, 95% confidence interval (CI) 0.46-0.72 and 0.47-0.72, respectively, *p* < 0.001). One-year OS and ORR rates were significantly improved in patients receiving either doses of pembrolizumab compared to ipilimumab as well (Table 2). The most common grade 3-5 AEs of special interest were colitis (1.4%, pembrolizumab every 2 weeks), colitis (2.5%) and hepatitis (1.8%, pembrolizumab every 3 weeks), and colitis (7.0%, ipilimumab) [16].

Furthermore, the FDA approved a labeling update for pembrolizumab in ipilimumab-refractory melanoma based on findings from KEYNOTE-002 [17]. This study compared pembrolizumab and investigator-choice chemotherapy (ICC) for the treatment of unresectable stage III or stage IV ipilimumab and/or BRAF inhibitor-refractory melanoma. Patients (*n* = 540) were randomized to receive pembrolizumab 2 mg/kg every 3 weeks, pembrolizumab 10 mg/kg every 3 weeks, or ICC (paclitaxel plus carboplatin, paclitaxel, carboplatin, dacarbazine, or temozolomide). There was no statistically significant difference in OS between both pembrolizumab arms and chemotherapy at interim analysis. Doses of pembrolizumab 2 mg/kg (HR 0.57, 95% CI 0.45-0.73, *p* < 0.001) and 10 mg/kg (HR 0.50, 95% CI 0.39-0.64, p < 0.001) showed superior median PFS when compared to chemotherapy. Response rates were 21% in the pembrolizumab 2 mg/kg group and 25% in the 10 mg/kg group compared to 4% in the chemotherapy arm (*p* < 0.0001). Incidence of grade 3-4 treatment-related AEs was higher in those given chemotherapy (26%) than in those given pembrolizumab 2 mg/kg group (11%) and pembrolizumab 2 mg/kg group (14%) [17].

### Nivolumab

On December 22, 2014, nivolumab was first approved as second-line treatment of unresectable or metastatic melanoma based on the CheckMate 037 trial [18]. This randomized controlled, open-label, international phase III study randomized 272 patients with unresectable stage IIIC or IV melanoma progressing after anti-CTLA-4 treatment or after anti-CTLA-4 treatment and a BRAF inhibitor for *BRAF*^*V600*^-mutated tumors to IV nivolumab 3 mg/kg every 2 weeks and 133 to ICC (Table 2). Positive PD-L1 expression was defined as ≥5% of tumor cells exhibiting PD-L1 staining (IHC 28-8 antibody) of any intensity in a section containing ≥100 evaluable cells. The ORR was 31.7% in the nivolumab group and 10.6% in the chemotherapy group (Table 2). In patients with PD-L1 positivity, ORR was 43.6% compared to 9.1% of the chemotherapy group. Grade ≥ 3 nivolumab-related AEs were seen in 9% of patients and included elevated lipase, elevated alanine aminotransferase (ALT), fatigue, and anemia. Grade ≥ 3 AEs occurred in 32% of chemotherapy patients, the most common of which were neutropenia, anemia, and thrombocytopenia.

The combination of nivolumab and ipilimumab was later approved as first-line treatment for *BRAF*^*V600*^-wild-type unresectable or metastatic melanoma on October 1, 2015 based on results from CheckMate 069 [19]. This randomized, double-blinded phase III trial, compared nivolumab 1 mg/kg in combination with ipilimumab 3 mg/kg (every 3 weeks X4 cycles then nivolumab alone every 2 weeks) against ipilimumab 3 mg/kg monotherapy (every 3 weeks) as first-line treatment in 142 patients with advanced melanoma. Objective response occurred in 61% of patients with *BRAF*^*V600*^-wild-type tumors in the combination group compared with 11% of patients in the monotherapy group. Of note, overall response was independent of PD-L1 status in both the combination group (58% for PD-L1-positive (≥5%) tumors vs. 55% for PD-L1-negative tumors) and the monotherapy group (18% for PD-L1- positive tumors and 4% for PD-L1 negative tumors). In patients with *BRAF*^*V600*^-mutated tumors, the ORR was 52% in the combination group compared with 10% in the monotherapy group. Grade ≥ 3 AEs occurred more frequently in the combination group (54%) than in the monotherapy group (24%), the most common of which were colitis, diarrhea, and elevated ALT. Ipilimumab monotherapy-related grade ≥ 3 AEs were seen in 24% of patients**,** the most common of which were diarrhea and colitis.

On January 23, 2016, nivolumab and ipilimumab combination therapy received an expanded approval for unresectable or metastatic melanoma irrespective of *BRAF*^*V600*^ mutation status based on results of the CheckMate 067 trial [20]. In this phase III trial, patients with untreated, unresectable or metastatic melanoma were randomized to receive nivolumab 3 mg/kg every 2 weeks, nivolumab 1 mg/kg and ipilimumab 3 mg/kg every 3 weeks for 4 doses followed by nivolumab 3 mg/kg every 2 weeks, or ipilimumab 3 mg/kg. Median PFS was 6.9 months in the nivolumab group, 11.5 months in the combination group, and 2.9 months in the ipilimumab group (Table 2). Longer OS was shown with nivolumab and combination therapy compared with ipilimumab alone across all subgroups (PD-L1 status, *BRAF*^*V600*^ status, and metastasis stage). The incidence of grade ≥ 3 AEs was greater in the combination group (55%) than in nivolumab or ipilimumab alone (16.3% and 27.3%, respectively). The most common grade ≥ 3 AEs in the combination group were diarrhea, colitis, and increased ALT and aspartate aminotransferase (AST) whereas the most frequent grade ≥ 3 AEs in the monotherapy arms were fatigue and diarrhea**.**

## Non-small cell lung cancer

### Pembrolizumab

On October 2, 2015, pembrolizumab was approved for treatment of previously-treated advanced or metastatic PD-L1-positive non-small cell lung cancer (NSCLC) [21]. As part of the KEYNOTE-001 phase I study, 550 patients were treated with either pembrolizumab at a dose of 2 mg/kg every 2 weeks or 10 mg/kg every 2 or 3 weeks (Table 3). The primary endpoints were antitumor activity per RECIST 1.1 and safety. Of the 61 patients with tumors identified as strongly positive for PD-L1 (PD-L1 ≥ 50% based on the companion diagnostic PD-L1 immunohistochemistry (IHC) 22C3 assay), the ORR for those receiving pembrolizumab 2 mg/kg was 28% (95% CI 12.1-49.4%) as compared to 40% (95% CI 22.4-61.2) and 41% (95% CI 24.7-59.3%) in patients receiving pembrolizumab 10 mg/kg every 2 weeks and every 3 weeks, respectively. The most commonly occurring (> 20%) AEs included fatigue, decreased appetite, dyspnea, and cough. Immune-mediated AEs occurred in 13% of patients and included pneumonitis, colitis, hypophysitis, and thyroid disorders [21].

**Table 3:** Registration trials leading to the FDA approval of PD-1/PD-L1 inhibitors in lung cancer

Study/Agent	Tumor (*n*)	Line of therapy	Experimental arm	Control arm	Primary endpoint	Ref
KEYNOTE-001 (phase Ib)/pembrolizumab	Advanced NSCLC (*n* = 550)	PD-L1 positive (≥1%) progressing after platinum-based therapy	Pembrolizumab 2 mg/kg every 3 weeks OR 10 mg/kg every 2 or 3 weeks		ORR 28% (95% CI 12.1-49.4%) vs. 40% (95% CI 22.4-61.2) vs. 41% (95% CI 24.7-59.3%) for PD-L1 ≥ 50% OS 22.1 mo (treatment-naïve, 95% CI 17.1-27.2) vs. 10.6 mo (previously-treated, 95% CI 8.6-13.3) for PD-L1 ≥ 50%	21, 22
KEYNOTE-024 (phase III)/pembrolizumab	Metastatic NSCLC with ≥50% PD-L1 expression (*n* = 305)	First-line	Pembrolizumab 200 mg every 3 weeks	ICC (cisplatin/carboplatin + pemetrexed, cisplatin/carboplatin + gemcitabine, or carboplatin + paclitaxel)	PFS 10.3 mos vs. 6.0 mos (HR 0.50, 95% CI 0.37-0.68, *p* < 0.001)	25
KEYNOTE-021 (phase II)/pembrolizumab	Advanced NSCLC (*n* = 123)	First line (in combination with platinum-doublet chemotherapy)	Pembrolizumab 200 mg + carboplatin AUC 5 mg/ml/min + pemetrexed 500 mg/m^2^ every 3 weeks X4 cycles followed by pembrolizumab (24 months duration) and indefinite maintenance pemetrexed	Carboplatin + pemetrexed X4 cycles followed by indefinite maintenance pemetrexed	ORR 55% vs. 29% (estimated treatment difference of 26%, 95% CI 9-42%, *p* = 0.0016)	26
CheckMate 017 (phase III)/nivolumab	Metastatic squamous NSCLC (*n* = 272)	Previously treated with platinum-based chemo	Nivolumab 3 mg/kg every 2 weeks	Docetaxel 75 mg/m^2^ every 2 weeks	OS 9.2 mo vs. 6.0 mos (HR 0.59, 95% CI 0.44-0.79, *p* < 0.001)	27
CheckMate 057 (phase III)/nivolumab	Metastatic non-squamous NSCLC (*n* = 582)	Previously treated with platinum-based chemo	Nivolumab 3 mg/kg every 2 weeks	Docetaxel 75 mg/m^2^ every 3 weeks	OS 12.2 mos vs. 9.4 mos (HR 0.73, 96% CI 0.59-0.89, *p* = 0.002)	28
POPLAR (phase II)/OAK (phase III)/atezolizumab	NSCLC (POPLAR *n* = 287, OAK *n* = 1225)	Second-line	Atezolizumab 1200 mg every 3 weeks	Docetaxel 75 mg/m^2^	POPLAR: OS 12.6 mos vs. 9.7 mos (HR 0.7, 95% CI 0.53-0.99, *p* = 0.04) OAK: OS 13.8 mos vs. 9.6 mos (HR 0.73, 95% CI 0.62-0.87, *p* = 0.0003)	29, 30

*FDA* Food and Drug Administration, *PD-1* programmed cell death 1, *PD-L1* programmed death ligand 1, *NSCLC* non-small cell lung cancer, *ORR* overall response rate, *CI* confidence interval, *OS* overall survival, *ICC* investigator-choice chemotherapy, *PFS* progression-free survival, *HR* hazard ratio, *AUC* area under curve

Updated long-term OS data for patients with previously-treated or treatment-naïve advanced or metastatic NSCLC were subsequently presented for the phase Ib KEYNOTE-001 study [22]. As compared to earlier studies that stratified tumor proportion score (TPS) cutoff of 1-50% and ≥50% PD-L1 staining of tumor cells, these investigators assessed a PD-L1 staining cutoff of ≥1% on tumor cells. Patients received either pembrolizumab 2 mg/kg every 3 weeks or 10 mg/kg every 2 or 3 weeks. Using a PD-L1 TPS cutoff of ≥1%, median OS was 22.1 months (95% CI 17.1-27.2) for treatment-naive patients and 10.6 months (95% CI 8.6-13.3) for previously-treated patients, supporting the efficacy of pembrolizuamb in patients with a PD-L1 TPS ≥1% [22]. KEYNOTE-001 investigators attempted to define a tumor PD-L1 expression level associated with an enhanced likelihood of benefit as well as validate the safety and antitumor activity of pembrolizumab in patients with advanced NSCLC and PD-L1 ≥ 50% expression [23]. Patients received pembrolizumab 2 mg/kg every 3 weeks or 10 mg/kg every 2 or 3 weeks and were randomized to either a training group or validation group. In the training group, the PD-L1 cutoff was selected by immune-related response criteria by investigator review; in the validation group, membranous PD-L1 expression ≥50% was selected as the cutoff. The ORR was 45.2% in the patients with PD-L1 ≥ 50%, including 43.9% in previously-treated patients and 50.0% in untreated patients. These values exceeded the response rate in the training group of 36.6%. Toxicities of grade ≥ 3 were reported in 47/495 patients (9.5%) and were most commonly dyspnea (3.8%), pneumonitis (1.8%), decreased appetite (1%), and asthenia (1%) [23].

Following KEYNOTE-001, KEYNOTE-010 was a phase II/III clinical trial that randomized 1034 patients to pembrolizumab (2 or 10 mg/kg every 3 weeks) vs. docetaxel (75 mg/m^2^) for PD-L1-positive NSCLC that progressed after platinum-based chemotherapy or a tyrosine kinase inhibitor (TKI) for those with an *EGFR*-sensitizing mutation or *ALK* gene rearrangement [24]. For patients with PD-L1 expression ≥1%, median OS for pembrolizumab 2 mg/kg (HR 0.71, 95% CI 0.58-0.88, *p* = 0.0008) and 10 mg/kg (HR 0.61, 95% CI 0.49-0.75, *p* < 0.0001) and median PFS for pembrolizumab 10 mg/kg (HR 0.79, 95% CI 0.66-0.94, *p* = 0.004) were significantly improved compared to docetaxel with a trend towards improved PFS with pembrolizumab 2 mg/kg. Pembrolizumab at both doses was superior to docetaxel in OS and PFS in those with ≥50% PD-L1 expression. Grade ≥ 3 treatment-related AEs occurred in 13% of the pembrolizumab 2 mg/kg group, 16% of the pembrolizumab 10 mg/kg group, and 35% of the docetaxel group. Deaths attributed to treatment occurred in 3 patients in the pembrolizumab 2 mg/kg group (2 of pneumonitis and 1 pneumonia), 3 patients in the pembrolizumab 10 mg/kg group (myocardial infarction, pneumonia, and pneumonitis), and 5 patients in the docetaxel group [24].

On October 24, 2016, pembrolizumab received approval as first-line treatment for metastatic NSCLC with ≥50% PD-L1 expression and without *EGFR* or *ALK* genomic tumor aberrations [25]. In the phase III KEYNOTE-024 trial, 305 patients were randomized to receive either pembrolizumab 200 mg every 3 weeks or ICC (platinum-based) for 4-6 cycles. Median PFS was 10.3 months in the pembrolizumab group as compared to 6.0 months in the chemotherapy group (HR 0.50, 95% CI 0.37-0.68, *p* < 0.001). Grade ≥ 3 treatment-related AEs occurred in 26.6% of the pembrolizumab group and 53.3% of the chemotherapy group [25].

On May 10, 2017, pembrolizumab received approval to be given in combination with pemetrexed and carboplatin as first-line treatment of metastatic NSCLC, irrespective of PD-L1 expression [26]. In the phase II KEYNOTE-021 open-label trial, 123 patients with stage IIIB or IV NSCLC who did not demonstrate targetable *EGFR* mutations or *ALK* translocations received either pembrolizumab 200 mg plus pemetrexed 500 mg/m^2^ and carboplatin area under the curve (AUC) 5 mg/mL/min every 3 weeks for 4 cycles follow by pembrolizumab 200 mg for 24 months and indefinite pemetrexed maintenance therapy, or pemetrexed 500 mg/m^2^ and carboplatin AUC 5 mg/mL/min followed by indefinite pemetrexed maintenance therapy alone. The primary endpoint ORR was 55% (33/60 patients) in the pembrolizumab plus chemotherapy group compared to 29% (18/63 patients) in the chemotherapy alone group, equating to an estimated treatment difference of 26% (95% CI 9-42%, *p* = 0.0016). The most common all-grade treatment-related AEs in the pembrolizumab arm vs. chemotherapy alone arm were fatigue (64% vs. 40%), nausea (58% vs. 44%), and anemia (32% vs. 53%).

### Nivolumab

Nivolumab was approved as treatment for metastatic squamous NSCLC on March 4, 2015 based on the CheckMate 017 trial [27]. In this phase III study, patients who progressed during or after 1 prior platinum-containing chemotherapy regimen were randomized to receive nivolumab 3 mg/kg every 2 weeks or docetaxel 75 mg/m^2^ every 3 weeks (Table 3). The primary endpoint was OS and a key secondary endpoint included efficacy based on tumor cell PD-L1 expression levels of 1%, 5% or 10%. Median OS was 9.2 months in the nivolumab group versus 6.0 months in the docetaxel group, and OS at 1 year was 42% in the nivolumab group versus 24% in the docetaxel group. PD-L1 expression was not predictive across any of the efficacy endpoints. Fewer all-grade treatment-related AEs occurred with nivolumab (58%) than with docetaxel (86%). The most frequently reported AEs were fatigue, decreased appetite, and asthenia with nivolumab compared to neutropenia, fatigue, and alopecia in the docetaxel arm. Grade ≥ 3 AEs were found in 7% of patients with nivolumab (including colitis and pneumonitis) compared 57% with docetaxel (including hemotologic toxicity and infections).

The CheckMate 057 trial ushered in the FDA-expanded approval of nivolumab in metastatic non-squamous NSCLC on October 9, 2015 [28]. This phase III trial enrolled 582 patients who had progressed during or after platinum-based doublet chemotherapy to receive nivolumab 3 mg/kg every 2 weeks or docetaxel 75 mg/m^2^ every 3 weeks. The primary endpoint was OS, which was 12.2 months with nivolumab and 9.4 months with docetaxel (Table 3). Treatment-related AEs occurred more frequently with docetaxel (20%) than nivolumab (7%). Grade ≥ 3 nivolumab-related AEs include fatigue, nausea, asthenia, and diarrhea; grade ≥ 3 docetaxel-related AEs included fatigue, anemia, and asthenia**.**

### Atezolizumab

On October 18, 2016, atezolizumab (PD-L1 inhibitor) was approved for previously-treated metastatic NSCLC following the results of the POPLAR and OAK trials [29, 30]. POPLAR is an ongoing phase II trial that randomized 287 patients to receive atezolizumab 1200 mg or docetaxel 75 mg/m^2^ with emphasis placed on PD-L1 expression of tumor cells and tumor-infiltrating immune cells [29]. The primary endpoint was OS and at a minimum follow-up of 13 months, atezolizumab had significantly improved OS compared with docetaxel (12.6 months vs. 9.7 months, *p* = 0.04). Increasing OS improvement was seen in subgroups with greater tumor cell and immune cell PD-L1 expression. However, unlike OS, improved PFS and ORR was limited to only those patients with the highest levels of PD-L1 expression (tumor cell ≥50% or immune cell ≥10%). The most common atezolizumab-related AEs were pneumonia and elevated AST levels.

Similarly, OAK is an ongoing phase III trial that randomized patients with previously-treated advanced NSCLC to atezolizumab 1200 mg every 3 weeks or docetaxel 75 mg/m^2^ every 3 weeks [30]. Patients were stratified by PD-L1 expression, number of previous chemotherapy regimens, and histology (squamous vs. non-squamous). OS was improved regardless of PD-L1 expression (Table 3) though patients with the highest PD-L1 expression experienced the greatest benefit from atezolizumab with a median OS of 20.5 months compared with 8.9 months in the docetaxel group. Grade ≥ 3 AEs were observed in 64% of patients in the atezolizumab cohort and included fatigue and anemia**.** Docetaxel-related grade ≥ 3 AEs were seen in 86% and were most frequently febrile neutropenia, neutropenia, anemia, and fatigue.

### Durvalumab

Although not FDA approved, it is worthwhile to mention that the PD-L1 inhibitor durvalumab was recently granted FDA breakthrough designation in the adjuvant treatment of locally advanced, unresectable NSCLC based on the phase III PACIFIC trial [31]. The primary endpoint was PFS, and 713 patients who did not demonstrate PD after ≥2 cycles of platinum-based chemotherapy concurrent with definitive RT were randomized to durvalumab (10 mg/kg) or placebo within 1-42 days after chemoradiotherapy every 2 weeks for up to 1 year. Durvalumab demonstrated superior PFS (median PFS 16.8 months, 95% CI 13.0-18.1) over placebo (5.6 months, 95% CI 4.6-7.8) in this setting (HR 0.52, 95% CI 0.42-0.65, *p* < 0.001). Safety was similar between both treatment arms with 29.9% of durvalumab patients and 26.1% of placebo patients experiencing grade 3-4 AEs. Improved outcomes were observed in the experimental arm irrespective of PD-L1 status or histology.

## Urothelial cancer

### Pembrolizumab

On May 18, 2017, pembrolizumab received 2 FDA approvals: in patients with locally advanced or metastatic urothelial carcinoma (UC) who have disease progression after platinum-containing chemotherapy and in patients who are cisplatin-ineligible [32, 33]. In the phase III, international KEYNOTE-045 trial, 542 patients with advanced UC showing ≥10% PD-L1 expression who had previously failed platinum-based chemotherapy were randomized to receive pembrolizumab 200 mg every 3 weeks or either paclitaxel, docetaxel, or vinflunine [33]. Median OS was significantly higher in the pembrolizumab group compared to chemotherapy though there was no significant difference in PFS (Table 4). Fewer grade ≥ 3 AEs occurred with pembrolizumab compared to the chemotherapy arm (15.0% vs. 49.4). Median OS was also significantly improved with pembrolizumab compared to chemotherapy (HR 0.57, 95% CI 0.37-0.88, *p* = 0.005) in those with PD-L1 expression ≥10% but there was no difference in PFS between arms in this population. In the phase II, open-label KEYNOTE-052 trial, patients with locally advanced or metastatic urothelial carcinoma who were cisplatin-ineligible received first-line pembrolizumab 200 mg every 3 weeks until progressive disease, unacceptable toxicity, or 24 months of treatment. The primary endpoint was ORR per RECIST 1.1. Of 370 enrolled patients, the ORR was 27% (95% CI 22-32) in those who had enrolled for ≥4 months. Grade ≥ 3 AEs occurred in 52 patients (14%) with 19 (5%) discontinuing therapy due to AEs [32].

**Table 4:** Registration trials leading to the FDA approval of PD-1/PD-L1 inhibitors in urothelial carcinoma and renal cell carcinoma

Study/Agent	Tumor (n)	Line of therapy	Experimental arm	Control arm	Primary endpoint	Ref.
KEYNOTE-052 (phase II)/pembrolizumab	Urothelial carcinoma (*n* = 370)	First-line cisplatin-ineligible	Pembrolizumab 200 mg every 3 weeks		ORR 24% (95% CI 20-29)	32
KEYNOTE-045 (phase III)/pembrolizumab	Urothelial carcinoma (*n* = 542)	Refractory to platinum-based chemotherapy	Pembrolizumab 200 mg every 3 weeks	Paclitaxel 175 mg/m^2^ OR docetaxel 75 mg/m^2^ OR vinflunine 320 mg/m^2^	OS 10.3 mos vs. 7.4 mos (HR 0.73, 95% CI 0.59-0.91, p = 0.002) PFS HR 0.98, 95% CI 0.81-1.19, *p* = 0.42	33
CheckMate 275 (phase II)/nivolumab	Advanced urothelial carcinoma (*n* = 270)	Previously treated with platinum-based chemotherapy	Nivolumab 3 mg/kg every 2 weeks		ORR 28.4% (95% CI 18.9-39.5) for 81 patients with PD-L1 ≥ 5%, 23.8% (95% CI 16.5-32.3) for 122 PD-L1 ≥ 1%, and 16.1% (95% CI 10.5-23.1) for 143 with PD-L1 < 1%	34
IMVigor 210 (phase II)/atezolizumab	Urothelial carcinoma (*n* = 315)	Previously treated with platinum-based chemotherapy	Atezolizumab 1200 mg every 3 weeks		ORR 27% (95% CI 19-37, *p* < 0.0001) for PD-L1 ≥ 5%, 18% (95% CI 13-24, *p* = 0.0004) for PD-L1 ≥ 1%, 15% (95% CI 11-20, *p* = 0.0058) for all patients compared to historical control	35
IMVigor 210 (phase II)/atezolizumab	Urothelial carcinoma (*n* = 119)	First-line cisplatin-ineligible	Atezolizumab 1200 mg every 3 weeks		ORR 23% (95% CI 16-31) in total population	36
Study 1108 (phase II)/durvalumab	Urothelial carcinoma (*n* = 191)	Second-line	Durvalumab 10 mg/kg every 2 weeks		ORR 17.8% (95% CI 12.7-24.0) in all patients, 27.6% (95% CI 19.0-37.5) for PD-L1 ≥ 25%, and 5.1% (95% CI 1.4-12.5) for PD-L1-negative	38
JAVELIN Solid Tumor (phase I)/avelumab	Urothelial carcinoma (*n* = 249)	Second-line	Avelumab 10 mg/kg every 2 weeks		ORR 17.4% (95% CI 11.9-24.1, complete response in 6.2%) for 61 post-platinum patients ≥6 months of follow-up	40
CheckMate 025 (phase III)/nivolumab	Advanced RCC (*n* = 821)	Second-line	Nivolumab 3 mg/kg every 2 weeks	Everolimus 10 mg daily	OS 25.0 mos vs. 19.6 mos (HR 0.73, 98.5% CI 0.57-0.93, *p* = 0.002)	41

*FDA* Food and Drug Administration, *PD-1* programmed cell death 1, *PD-L1* programmed death ligand 1, *ORR* overall response rate, *CI* confidence interval, *OS* overall survival, *HR* hazard ratio, *PFS* progression-free survival, *RCC* renal cell carcinoma

### Nivolumab

The FDA approved nivolumab on February 2, 2017 for locally advanced or metastatic UC following the results from CheckMate 275 [34]. This phase II study enrolled 270 patients who had experienced progression or recurrence after ≥1 platinum-based chemotherapy regimen to receive nivolumab 3 mg/kg every 2 weeks (Table 4). The primary endpoint was ORR in all treated patients stratified by PD-L1 expression (28.4% for ≥5%, 23.8% for ≥1%, and 16.1% for < 1%). At median follow up of 7 months, OS was 11.30 months in the PD-L1 ≥ 1% subgroup and 5.95 months in the PD-L1 < 1% subgroup. Grade 3-4 AEs related to nivolumab included diarrhea and fatigue.

### Atezolizumab

On May 18, 2016, atezolizumab became the first PD-L1 inhibitor approved for locally advanced and metastatic UC based on results of IMVigor 210 [35]. This phase II trial enrolled 310 patients whose disease had progressed after receiving platinum-based chemotherapy to receive a fixed dose of atezolizumab 1200 mg every 3 weeks (Table 4). PD-L1 status was subdivided by the percentage of PD-L1-positive immune cells in the tumor microenvironment (TME): < 1%, ≥1% but < 5%, and ≥5%. The primary endpoint was ORR. In all patients, ORR was 15%, a significant improvement compared to the historical response rate of 10%. In addition, subgroup analysis showed a PD-L1-related response: PD-L1 ≥ 5% showed a 27% ORR, PD-L1 ≥ 1% showed 18% ORR, and PD < 1% showed 8% response. Sixteen percent of patients experienced grade 3-4 treatment-related AEs, the most common of which were fatigue, anemia, and hypertension. Notably a recent press release for the confirmatory IMVigor 211 trial reported a failure to meet the study’s primary endpoint (see Discussion).

Accelerated approval of atezolizumab in the first-line treatment of cisplatin-ineligible patients with locally advanced and metastatic UC occurred based on a separate cohort of the IMVigor 210 trial [36]. This phase II, single-arm trial administered atezolizumab 1200 mg every 3 weeks to 119 treatment-naïve metastatic UC with stratification based on PD-L1 expression as in the earlier IMVigor 210 trial. The primary endpoint was independently confirmed ORR per RECIST v1.1. In the primary analysis, efficacy did not reach (PD-L1 ≥ 5% subgroup) the pre-specified ORR of 10% after a median follow-up of 8.5 months. After a 17.2 month median follow up duration, the ORR increased to 28% in the PD-L1 ≥ 5% subgroup, 21% in the ≥1% PD-L1 but < 5% group, and 21% in the PD-L1 < 1% group. Interestingly, median OS was 15.9 months in all patients, 12.3 months in PD-L1 ≥ 5% patients, and 19.1 months in PD-L1 < 5% patients. The most common grade 3-4 treatment-related AEs were fatigue and elevated AST and ALT.

### Durvalumab

Durvalumab received FDA approval on May 1, 2017 for the treatment of platinum-refractory locally advanced or metastatic UC based on results from Study 1108 [37]. In this phase I/II dose-escalation and expansion study, 61 patients who had progressed on, been ineligible for, or refused prior therapies for advanced disease were enrolled to receive the PD-L1 inhibitor durvalumab 10 mg/kg of every 2 weeks. Patients were initially enrolled regardless of PD-L1 expression, but enrollment was later restricted to patients with ≥5% PD-L1 expression on tumor cells after preliminary data suggested PD-L1 was expressed more commonly on immune cells than tumor cells. The primary endpoint was safety and of 42 treated patients, grade ≥ 3 AEs occurred in 3 patients. Of note, the ORR was 31.0% in all 42 patients, 46.4% in the PD-L1-positive subgroup, and 0% in the PD-L1-negative subgroup.

In an update of Study 1108, results were presented regarding the efficacy and tolerability of durvalumab 10 mg/kg every 2 weeks up to 12 months [38]. High PD-L1 expression was defined as ≥25% of tumor or immune cells (Ventana SP263 assay). The primary endpoint was ORR using RECIST 1.1 (Table 4). Of the 191 treated patients, ORR was 17.8% (95% CI 12.7-24.0) in all patients, 27.6% (95% CI 19.0-37.5) for PD-L1 ≥ 25%, and 5.1% (95% CI 1.4-12.5) for PD-L1-negative. Grade 3-4 AEs related to treatment were seen in only 6.8% of patients.

### Avelumab

Avelumab (PD-L1 inhibitor) received accelerated approval for locally advanced or metastatic UC following the JAVELIN Solid Tumor study [39]. In this phase Ib study, 44 patients with metastatic or locally advanced solid tumors after platinum-based therapy were given escalating doses of avelumab 10 mg/kg every 2 weeks. The primary endpoint was safety and 1 dose-limiting toxicity was reported at dose level 4 in a patient with metastatic thymoma who developed autoimmune disorder and increased blood creatine phosphokinase (CPK). Grade 3-4 treatment-related AEs occurred in 3 patients (6.8%) and included asthenia, AST elevation, elevated CPK, and decreased appetite.

In the phase Ib update to the JAVELIN Solid Tumor study (Table 4), dose-expansion occurred up to 249 patients with metastatic UC refractory to platinum-based therapy or ineligible for cisplatin-therapy [40]. In 161 post-platinum patients with ≥6 months of follow-up, responses were seen across PD-L1 expression levels tested (≥5% and < 5% PD-L1 tumor cell-staining (25.4% and 13.2%, respectively). Immune-related AEs occurred in 34 pts. (13.7%) with an incidence of grade ≥ 3 events in 2.4%.

## Renal cell carcinoma

### Nivolumab

On November 23, 2015, nivolumab became the first PD-1 inhibitor approved for use in treatment-refractory clear-cell renal cell carcinoma (RCC) based on results from CheckMate 025 [41]. In this phase III study, 821 patients were randomized to receive nivolumab 3 mg/kg every 2 weeks or oral everolimus 10 mg daily (Table 4). The primary endpoint was OS, which was 25.0 months with nivolumab and 19.6 months with everolimus. Of note, patients with ≥1% PD-L1 expression demonstrated median OS of 21.8 months with nivolumab and 18.8 months with everolimus. Similar results were seen in patients with ≥5% PD-L1 expression, though interpretation is limited by the small sample size in this subgroup. The most frequent grade ≥ 3 AEs were fatigue with nivolumab and anemia with everolimus (19% and 37%, respectively).

## Head and neck cancer

### Pembrolizumab

On August 5, 2016, pembrolizumab received accelerated approval for recurrent or metastatic HNSCC with disease progression on or after platinum-containing chemotherapy [42]. The KEYNOTE-012 open-label, multicenter, phase Ib trial studied the efficacy and safety of pembrolizumab in patients with ≥1% of tumor cells that were PD-L1-positive. Sixty patients received pembrolizumab 10 mg/kg every 2 weeks for 24 months and the primary endpoints were safety and ORR per RECIST 1.1 (Table 5). The ORR was 18% (95% CI 8-32%) and 10 (16.7%) experienced grade ≥ 3 AEs with the most common being transaminitis, hyponatremia, and rash.

**Table 5:** Registration trials leading to the FDA approval of PD-1/PD-L1 inhibitors in head and neck cancer, classical Hodgkin lymphoma, colorectal cancer, gastroesophageal cancer, hepatocellular cancer, and other solid cancers

Study/Agent	Tumor (n)	Line of therapy	Experimental arm	Control arm	Primary endpoint	Ref.
KEYNOTE-012 (phase Ib)/pembrolizumab	HNSCC (*n* = 60)	PD-L1 ≥ 1% and refractory to platinum chemotherapy	Pembrolizumab 10 mg/kg every 2 weeks		Safety 45% with serious AEs, 17% with grade 3-4 AEs (most common transaminitis, hyponatremia, and rash) ORR 18% (95% CI 8-32%)	42
CheckMate 141 (phase III)/nivolumab	HNSCC (*n* = 361)	Previously treated with platinum-based chemotherapy	Nivolumab 3 mg/kg every 2 weeks	ICC: either weekly cetuximab 250 mg/m^2^ after a loading dose of 400 mg/m^2^, weekly methotrexate 40-60 mg/m^2^, or weekly docetaxel 30-40 mg/m^2^	OS 7.5 mos vs. 5.1 mos (HR 0.70, 97.73% CI 0.51-0.96, *p* = 0.01)	43
KEYNOTE-087 (phase II)/pembrolizumab	cHL (n = 210)	Relapsed after ≥3 lines of therapy or refractory cHL	Pembrolizumab 200 mg every 3 weeks		ORR 69.0% (95% CI 62.3-75.2%) CR 22.4% (95% CI 16.9- 28.6%)	44
CheckMate 039 (phase I), CheckMate 205 (phase II)/nivolumab	cHL (*n* = 80)	Previously treated with ASCT or brentuximab	Nivolumab 3 mg/kg every 2 weeks		ORR 66.3% (95% CI 54.8-76.4)	45, 46
Five phase I and II trials (including KEYNOTE-164 and KEYNOTE-158)/pembrolizumab	MSI-H or dMMR unresectable or metastatic solid tumors (*n* = 149 across five trials)	Treatment-refractory to all standard therapies	Pembrolizumab 200 mg every 3 weeks		ORR 39.6%	47-53
KEYNOTE-059 (phase II)/pembrolizumab	Advanced gastric or gastroesophageal junction cancer (*n* = 259)	PD-L1 ≥ 1% and progression on ≥2 lines of chemotherapy	Pembrolizumab 200 mg every 3 weeks		ORR 11.2% (95% CI 7.6-15.7)	54
CheckMate 142 (phase II)/nivolumab	Metastatic colorectal cancer (*n* = 74)	Previously treated with fluoropyrimidine, oxaliplatin, and irinotecan	Nivolumab 3 mg/kg every 2 weeks		ORR 31.1% (95% CI 20.8-42.9)	55
CheckMate 040 (phase 1/2)	Advanced hepatocellular carcinoma (*n* = 262)	Refractory to one previous line of therapy (including sorafenib), or intolerant of sorafenib	Nivolumab 3 mg/kg every 2 weeks		Safety 12/48 patients (25%) grade 3-4 AEs with 3 (6%) having treatment-related serious AEs (pemphigoid, adrenal insufficiency, liver disorder) ORR 20% (95% CI 15-26%)	56
JAVELIN Merkel 200 (phase II)	Merkel cell carcinoma (*n* = 88)	First-line and beyond	Avelumab 10 mg/kg every 2 weeks		ORR 31.8% (95.9% CI 21.9-43.1)	57

*FDA* Food and Drug Administration, *PD-1* programmed cell death 1, *PD-L1* programmed death ligand 1, *HNSCC* head and neck squamous cell carcinoma, *AEs* adverse events, *ORR* overall response rate, *CI* confidence interval, *ICC* investigator-choice chemotherapy, *OS* overall survival, *HR* hazard ratio, *cHL* classical Hodgkin lymphoma, *CR* complete response, *ASCT* autologous stem cell transplantation, *MSI-H* microsatellite instability-high, *dMMR* defective mismatch repair

### Nivolumab

On November 10, 2016, nivolumab became the first immunotherapy approved by the FDA for HNSCC based on results from CheckMate 141 [43]. This phase III trial randomized 361 patients with disease that recurred or progressed within 6 months of the last dose of platinum-containing chemotherapy to nivolumab 3 mg/kg every 2 weeks or ICC (Table 5). The primary endpoint was OS, which was 7.5 months with nivolumab and 5.1 months with ICC. Estimated 6-month PFS rates were 19.7% (nivolumab) and 9.9% (ICC). Grade ≥ 3 nivolumab-related AEs occurred in 13% and included fatigue, anemia, asthenia, and stomatitis. Grade ≥ 3 chemotherapy-related AEs were seen in 35% and most commonly were anemia and neutropenia.

## Hodgkin lymphoma

### Pembrolizumab

On March 15, 2017, pembrolizumab received approval for a hematologic malignancy based on the findings from the KEYNOTE-087 trial (Table 5) [44]. Patients (*n* = 210) with relapsed or refractory classical Hodgkin Lymphoma (cHL from 3 cohorts: 1.) after autologous stem cell transplantation (ASCT) and subsequent brentuximab vedotin (BV), 2.) after salvage chemotherapy and BV with chemoresistant disease, and 3.) after ASCT but without BV after transplantation received pembrolizumab 200 mg every 3 weeks for a maximum of 24 weeks. The ORR was 73.9% for cohort 1, 64.2% for cohort 2, and 70.0% for cohort 3. The most common grade ≥ 3 treatment-related AE was neutropenia (2.4%).

### Nivolumab

On May 17, 2016, nivolumab received the first approval for a PD-1 inhibitor in the treatment of a hematologic malignancy based on the findings from CheckMate 039 and CheckMate 205 (Table 5) [45, 46]. CheckMate 039 was a phase I study consisting of dose-escalation and expansion cohorts of patients with relapsed or refractory hematologic cancers treated with nivolumab 1 mg/kg with escalation to 3 mg/kg, and patients in the expansion cohort received nivolumab 3 mg/kg at week 1, week 4, and every 2 weeks up to 2 years [45]. The primary endpoint was safety, and of the 23 patients with cHL enrolled, grade ≥ 3 AEs were seen in 5 patients including myelodysplastic syndrome, pancreatitis, and pneumonitis. Results of this trial showed promising efficacy of nivolumab in cHL.

The CheckMate 205 trial was a phase II study enrolling 80 patients with cHL who had relapsed after ASCT or BV to receive nivolumab 3 mg/kg every 2 weeks [46]. The primary endpoint was independently assessed ORR and was 66.3% (53/80 patients) with 52 of the 53 responders having > 50% tumor reduction. Notably, in a post-hoc analysis of patients who did not have response to BV as the most recent treatment prior to trial recruitment, 31 of 43 patients achieved objective response after nivolumab treatment. Grade ≥ 3 AEs were seen in 25%, the most frequent of which were increased lipase and neutropenia.

## Microsatellite instability or mismatch repair deficient cancers

### Pembrolizumab

In the first tissue-agnostic indication for a therapeutic agent, pembrolizumab was approved on May 23, 2017 (Table 5) for patients with treatment-refractory unresectable or metastatic solid tumors that are microsatellite instability-high (MSI-H) or mismatch repair deficient (dMMR) [47–53]. Two phase 2 studies have showed ORR of 48% in 29 patients and 50% in 10 patients with various dMMR cancers, while a pivotal phase 2 study identified an ORR of 40% in 10 dMMR colorectal cancer (CRC) patients and an ORR of 71% in 7 dMMR non-CRC patients [48, 52, 53]. As part of the ongoing, global, multicenter phase II studies KEYNOTE-164 and KEYNOTE-158, the ORR was 26.2% for 61 MSI-H CRC patients and 42.9% for 21 MSI-H non-CRC patients [47]. In 2 trials evaluating the role pembrolizumab in dMMR tumors, ORR was 50% in 28 dMMR CRC patients and 53% in 78 patients with various dMMR tumors [50, 51]. Another single-institution phase II study reported an ORR of 56% in 9 patients with dMMR endometrial cancer [49].

## Gastric cancer

### Pembrolizumab

Recently on September 22, 2017, pembrolizumab 200 mg every 3 weeks was approved for advanced gastroesophageal cancer that is PD-L1 ≥ 1% (IHC 22C3 antibody) and refractory ≥2 lines of chemotherapy based on the phase II KEYNOTE-059 trial [54]. Out of 259 patients, the ORR was 11.2% (95% CI 7.6-15.7) with a median duration of response of 8.1 months (Table 5). Grade 3-5 treatment-related AEs occurred in 43 patients (16.6%) leading to discontinuation in 2 patients and death in 2 patients due to renal failure and pleural effusion.

## Colorectal cancer

### Nivolumab

On August 1, 2017, nivolumab was approved in dMMR/MSI-H metastatic colorectal cancer (mCRC) refractory to fluoropyrimidine, oxaliplatin, and irinotecan [55]. This approval was granted based on results from the CheckMate 142 trial, a phase II trial in which patients received nivolumab 3 mg/kg every 2 weeks and were stratified by PD-L1 < 1% and PD-L1 ≥ 1%. The primary endpoint was ORR per RECIST 1.1. Of the 74 patients enrolled, 23 patients (31%) achieved ORR irrespective of PD-L1 levels (Table 5). Nivolumab-related grade ≥ 3 AEs occurred in 12% of patients, most commonly fatigue, diarrhea, and pruritus.

## Hepatocellular carcinoma

### Nivolumab

Recently on September 22, 2017, nivolumab 3 mg/kg every 2 weeks was approved in advanced hepatocellular carcinoma (HCC) refractory to sorafenib in the phase I/II CheckMate 040 trial [56]. Of 262 eligible patients, ORR was 20% (95% CI 15-26%) with no maximum-tolerated dose established in the dose-escalation phase. Activity and tolerability did not appear to be affected by PD-L1 status or presence or absence of viral hepatitis (Table 5). Twelve of 48 patients (25%) experienced grade 3-4 AEs with 3 patients (6%) having treatment-related serious AEs (pemphigoid, adrenal insufficiency, liver disorder).

## Merkel cell carcinoma

### Avelumab

Avelumab, a fully humanized monoclonal IgG1 antibody against PD-L1, was first approved on March 23, 2017 for treatment of metastatic Merkel cell carcinoma (untreated and chemotherapy-resistant). This approval was granted based on the results of the JAVELIN trial, a single-arm phase II trial in which patients with stage 4 Merkel cell carcinoma refractory to ≥1 previous line of chemotherapy received IV avelumab 10 mg/kg every 2 weeks [57]. The primary endpoint was ORR (Table 5). Complete response was observed in 9% of patients and partial response observed in 23%, at a median follow-up time of 10.4 months. Among the patients whose tumors were assessable for PD-L1 expression (with PD-L1 positivity defined as a threshold level of 1% positive cells of any intensity), 34.5% (95% CI, 22.5-48.1) achieved objective responses. Grade ≥ 3 toxicities were reported in 5% of patients including lymphopenia and isolated laboratory abnormalities.

## Discussion

Since the FDA approvals of the first PD-1 inhibitors pembrolizumab and nivolumab in 2014, the clinical development of PD-1 and PD-L1 inhibitors as anticancer agents has picked up considerable momentum [13–15, 18]. There are currently 5 PD-1/PD-L1 inhibitors that are FDA-approved in the treatment of a number of solid tumors (Tables 1, 2, 3, 4 and 5). Approved indications in this class of immune checkpoint inhibitors have also expanded to include hematologic malignancies and specific molecular phenotypes irrespective of solid tumor histology (i.e., tissue-agnostic) [45–53, 55]. As the number of PD-1/PD-L1 inhibitors undergoing development is expected to rise in the foreseeable future, several important points of discussion need to be considered in order to optimize the anticancer potential of this class of agents.

### Predictive biomarkers

Despite the promising anticancer activity offered by PD-1 and PD-L1 inhibitors, predicting tumor responses to PD-1/PD-L1 blockade remains a challenge given that not all patients derive benefit from this class of immunotherapy. Perhaps the earliest and most widely recognized predictive biomarker of response to PD-1/PD-L1 blockade is PD-L1 expression, for which there are 4 FDA-approved assays of PD-L1 expression by IHC (Table 1) to help guide treatment decisions for nivolumab in advanced NSCLC or melanoma (Dako 28-8), pembrolizumab in advanced NSCLC (Dako 22C3), atezolizumab in advanced urothelial carcinoma or NSCLC (Ventana SP142), and durvalumab in advanced urothelial carcinoma (Ventana SP263) [20, 24, 27, 28, 35, 37, 38]. A recent meta-analysis involving 41 clinical trials and 6664 patients with advanced solid tumors investigated the predictive value of tumor and tumor-infiltrating immune cell PD-L1 expression by IHC assays such as Dako 28-8, Dako 22C3, Ventana SP142, Ventana SP263, and Dako clone 73-10 and demonstrated that PD-L1 expression was predictive of tumor response across all tumor types (odds ratio (OR) 2.26, 95% confidence interval (CI) 1.85-2.75, *p* < 0.001) [58]. Of note, the largest effect reaching significance was observed in NSCLC (OR 2.51, 95% CI 1.99-3.17, p < 0.001). However, despite the promising utility of PD-L1 expression as a biomarker for PD-1/PD-L1 blockade, there is growing concern regarding its true predictability for response given its highly variable, heterogeneous, and dynamic expression on tumor or tumor-infiltrating immune cells [12]. Furthermore, technical differences and variation in screening thresholds for PD-L1 expression across assays represent additional limitations. This was shown in a recent multi-institutional collaborative effort to provide information on the analytic comparability of the 4 FDA-approved IHC assays of PD-L1 expression (22C3, 28-8, SP142, and SP263) [59]. Out of 39 NSCLC tumors stained, 3/4 assays showed a comparable percentage of PD-L1-stained tumor cells while the SP142 assay showed fewer stained tumor cells overall. There was greater variability in immune cell staining than tumor cell staining across all 4 assays. Notably, in 14/38 cases (37%) a different PD-L1 classification would have been made depending on which IHC assay and scoring system was used. A larger Phase II effort is currently underway to validate these findings. Nevertheless, although PD-L1 expression is associated with a higher likelihood of response to PD-1/PD-L1 blockade, it has yet to be proven as the definitive biomarker for efficacy and its absence certainly does not preclude response to PD-1/PD-L1 inhibitors.

The search for the ideal biomarker of response to PD-1/PD-L1 blockade is undergoing active investigation. There is increasing evidence to support that high mutational load can predict benefit from immune checkpoint inhibitors across several tumor types due to the immunogenic nature of neoantigens generated from an increased burden of nonsynonymous mutations [60]. For example, MSI or dMMR tumors are predisposed to accumulation of frameshift mutations due to defective DNA repair machinery and have shown significantly greater responses to PD-1 blockade compared to microsatellite stable (MSS) or mismatch repair-proficient tumors [47–53, 55]. Tumors harboring *POLE* mutations represent another phenotype with high tumor mutational burden that may predict response to PD-1 blockade [60, 61]. Other investigations have focused on the presence of an immune-active TME. Here, a TME associated with higher densities of CD8+ tumor-infiltrating lymphocytes (TILs) with a Th1 phenotype and more clonal T-cell receptor (TCR) repertoire, higher levels of interferon (IFN), IFN-γ-inducible genes, and IFN-stimulated chemokines such as CXCL9, CLCL10, and CXCL11, and high levels of immune checkpoints such as cytotoxic T-lymphocyte antigen 4 (CTLA-4), PD-L1/PD-L2, PD-1, and indoleamine 2,3-dioxygenase (IDO) may predict benefit from anti-PD-1 and anti-PD-L1 therapy [12, 60, 62]. In contrast to the immunologically “hot” TME, “cold” or non-T-cell-inflamed tumors have been associated with activated Wnt/β-catenin pathway signaling and *PTEN* deficiency [60, 62]. Features that define an immunologically hot or T-cell-inflamed tumor are becoming increasingly complex with evidence to support a role for CD4+ T-cells, T-regulatory cells, and myeloid-derived suppressor cells in contributing to a TME that is responsive to PD-1/PD-L1 blockade [60, 62].

Lastly, genetic polymorphisms and composition of the gut microbiome may also shape an individual’s potential to respond to immune checkpoint inhibitors, and prospective studies are underway to investigate these novel concepts [12, 60, 62]. Significant differences in baseline diversity and composition of the gut microbiome between responders and nonresponders to anti-PD-1 therapy in metastatic melanoma patients have been reported, with enrichment of the Ruminococcaceae family of the *Clostridiales* order in responders whereas the Prevotellaceae family of the *Bacteroidales* order was enriched in nonresponders [63]. Other studies in melanoma mice models have identified that commensal gut bacteria such as *Bifidobacterium* putatively enhance response to anti-PD-L1 therapy by modulating immune responses through T-cell regulatory pathways [64]. Conversely, antibiotics can affect 30% of gut microbiota, and retrospective analyses in advanced solid tumor patients treated with anti-PD-1/PD-L1 therapy showed that receipt of antibiotics prior to immunotherapy was a negative predictor of survival on multivariate analysis [65]. Future directions of investigation may seek to explore the utility of a comprehensive assessment that takes into account features of the TME and other immune parameters to produce a composite score predictive of benefit to PD-1/PD-L1 blockade; one such tool, the Immunoscore, has already been demonstrated as a strong prognostic indicator in CRC with potential to guide immunotherapy strategies [66].

### Mechanisms of resistance and hyperprogressors

Blockade of the PD-1/PD-L1 axis results in antitumor activity due to its ability, in part, to inhibit interferon-induced adaptive immune resistance characterized by interferon-induced JAK-STAT signaling that results in activation of interferon regulatory factor 1 (IRF1) and expression of PD-L1 and IDO that allow for cancer cell immune evasion [67]. Innate resistance to anti-PD-1 therapy has been characterized by upregulation of genes involved in the regulation of cell adhesion, extracellular matrix remodeling, mesenchymal transition, angiogenesis, and wound healing [68]. Acquired resistance to checkpoint blockade has been characterized by loss of sensitivity to IFN-γ either through mutations or epigenetic silencing of mediators of the IFN-γ//JAK/STAT/IRF1 signaling pathway [67, 69]. In addition, one study was among the first to describe the existence of a subset of patients (9%) experiencing hyperprogressive disease defined as RECIST progression at first evaluation characterized by a ≥ 2-fold increase in tumor growth rate in response to anti-PD-1/PD-L1 therapy [70]. This novel pattern of hyperprogression was associated with higher age and worse OS. In a separate study, tumors from 155 patients with advanced cancers treated with PD-1/PD-L1 inhibitors were evaluated by next-generation sequencing to evaluate potential genomic markers associated with hyperprogressive disease defined as time-to-treatment failure (TTF < 2 months, > 50% increase in tumor burden compared to pre-immunotherapy imaging, and > 2-fold increase in progression pace [71]. Hyperprogessors to single-agent PD-1/PD-L1 blockade were found to have *MDM2* family amplifications or *EGFR* aberrations that significantly correlated to a TTF < 2 months on multivariate analysis.

Further understanding of mechanisms of resistance and identification of hyperprogressors are certainly warranted in large, prospective cohorts to optimize efficacy and minimize risk to PD-1/PD-L1 inhibitors. Moreover, given the complexities of immunoregulatory pathways and host and tumor heterogeneity, combination strategies incorporating PD-1/PD-L1 blockade with vaccines, radiation therapy, stimulators of T-cell activity through targeting of CD40/CD40L, OX40/OX40L, and 4-1BB (CD137), co-targeting of other immune checkpoints such as T-cell immunoglobulin mucin 3 (Tim-3), lymphocyte activation gene 3 protein (LAG3), IDO, and T-cell immunoglobulin and ITIM domain (TIGIT), adoptive T-cell therapy, epigenetic reprogramming drugs, chemotherapy, and targeted agents such as vascular endothelial growth factor (VEGF)-directed therapy are increasingly being employed in clinical trials to enhance sensitivity to immunotherapy [62].

### Immune-related adverse events

Paramount to the safe and effective administration of anti-PD-1 and anti-PD-L1 therapy is our greater recognition and understanding of their potential immune-related toxicities. A recent meta-analysis of 3450 patients receiving PD-1/PD-L1 inhibitors demonstrated higher risk of all-grade rash, pruritus, hypothyroidism, hyperthyroidism, colitis, aminotransferase elevations, and pneumonitis but lower risk of all-grade AEs in general and lower risk of all-grade fatigue, sensory neuropathy, diarrhea, hematologic toxicities, anorexia, nausea, and constipation, and treatment discontinuation when compared to chemotherapy [72]. Nevertheless, immune-related toxicities can often be nontrivial resulting in significant risks that outweigh potential benefits of PD-1/PD-L1 inhibitors. For example, beginning July 2017, the FDA has placed clinical holds on several clinical trials investigating pembrolizumab-, nivolumab-, and durvalumab-containing regimens in various hematologic malignancies based on findings and safety concerns identified from the KEYNOTE-183 and KEYNOTE-185 studies [73–75]. A detailed description of specific immune-related AEs and their management is beyond the scope of this review and has been extensively reviewed elsewhere; however, there is growing evidence that reassuringly shows use of systemic immunosuppressants may not negatively impact outcomes derived from checkpoint blockade [76–80].

### Treatment duration, treatment beyond progression, and response after prior PD-1/PD-L1 blockade

The optimal duration of treatment with PD-1/PD-L1 inhibitors remains undefined but is of increasing relevance given the potential for delayed responses and the uncommon but documented phenomenon of pseudoprogression with immune checkpoint inhibitors [81]. Many randomized clinical trials investigating anti-PD-1 therapy across several tumor types have allowed for treatment beyond first progression (TBP) provided that patients continued to exhibit investigator-assessed clinical benefit, stable performance status, and tolerance to therapy without substantial adverse effects [81]. Available post hoc subgroup analyses of these trials have demonstrated that 9-48% of patients received TBP ≥4 or 6 weeks with anti-PD-1 therapy, and of these, 13-33% of patients experienced > 30% target lesion reduction after progression when compared to baseline imaging [81–85]. Compared to non-TBP patients, TBP patients often showed improved PFS and OS though often with higher incidence of treatment-related AEs consistent with prolonged exposure to anti-PD-1 therapy. It remains unclear, however, whether patients who experienced additional benefit with TBP had contributing factors such as better prognostic features and likely more indolent disease to begin with and whether the small subset of the overall population of patients that benefit from TBP is worth the increased toxicity, increased cost, and risk of delaying alternative and more effective therapies in choosing this approach [81]. Furthermore, many randomized clinical trials have employed conventional RECIST criteria to assess the efficacy of PD-1 inhibitors [82–86]. The novel iRECIST criteria has recently been proposed to allow more consistent interpretation of response and progression to cancer immunotherapy [87]. For the question of response to PD-1/PD-L1 blockade after prior treatment with PD-1/PD-L1 inhibitors, evidence is limited but appears to support an unlikely response with subsequent treatment in this scenario; there are, however, numerous ongoing and pending prospective clinical trials involving PD-1/PD-L1 blockade that allow prior treatment with PD-1/PD-L1 inhibitors that may provide more information on this topic [88]. Future studies of ideally prospective design are warranted to address remaining questions on optimal duration, TBP vs. switching to agents of a different class on progression, and treatment to progression or best response followed by rechallenge with PD-1/PD-L1 inhibitors.

### Clinical trial design

Lastly, several confirmatory phase III trials KEYNOTE-040, IMVigor211, and CheckMate 026 have failed to meet their primary endpoints of PFS or OS despite promising results in prior studies that in some instances resulted in earlier FDA approval [89–91]. Differences in patient selection and baseline characteristics, variation among biomarker assays and PD-L1 expression cut-off thresholds, sampling for PD-L1 expression on metastatic lesions vs. archival tissue biopsy, subsequent immunotherapy in the standard of care arms, and outperformance or overachievement of study assumptions by standard of care therapies have been among the many, but not all, potential explanations for these recent results [89, 90, 92, 93]. There is curiosity regarding the fate of FDA-labeled indications for specific PD-1/PD-L1 inhibitors that were earlier approved but dependent on confirmatory phase III trials. Nevertheless, these negative trials highlight the importance of all aspects of clinical trial design in evaluating the efficacy of immune checkpoint inhibitors and provide invaluable learning for subsequent confirmatory trials. Furthermore, others have proposed implementation of iRECIST criteria and incorporation of weighted-log rank tests into future study designs as considerations to improve our interpretability of success or failure with PD-1/PD-L1 inhibitors [87, 94].

## Conclusions

Since the FDA approvals of the first PD-1 inhibitors pembrolizumab and nivolumab in 2014, the clinical development of PD-1/PD-L1 inhibitors as a form of cancer immunotherapy has seen unprecedented growth. There are currently 5 PD-1/PD-L1 inhibitors that are approved for the treatment of a spectrum of cancers including hematologic malignancies. As the number of anti-PD-1 and anti-PD-L1 therapies is expected to rise in the foreseeable future, there are several key issues that remain and require further investigation in order to optimize the anticancer potential of this class of agents. Specifically, predictive biomarkers, mechanisms of resistance, immune-related toxicities, hyperprogressors, treatment duration and TBP, and clinical trial design represent areas in need of further consideration to optimize benefit and minimize risks from PD-1/PD-L1 blockade.

## Abbreviations

AEs: Adverse events
ALT: Alanine aminotransferase
ASCT: Autologous stem cell transplantation
AST: Aspartate aminotransferase
AUC: Area under the curve
BV: Brentuximab vedotin
cHL: Classical Hodgkin lymphoma
CI: Confidence interval
CPK: Creatine phosphokinase
CRC: Colorectal carcinoma
CTLA-4: Cytotoxic T-lymphocyte antigen 4
dMMR: Mismatch repair deficient
FDA: Food and drug administration
HCC: Hepatocellular carcinoma
HNSCC: Head and neck squamous cell carcinoma
HR: Hazard ratio
ICC: Investigator-choice chemotherapy
IDO: Indoleamine 2,3-dioxygenase
IFN: Interferon
IHC: Immunohistochemistry
IRF1: Interferon regulatory factor 1
IV: Intravenous
LAG3: Lymphocyte activation gene 3 protein
MSI-H: Microsatellite instability-high
MSS: Microsatellite stable
NSCLC: Non-small cell lung cancer
ORR: Overall response rate
OS: Overall survival
PD-1: Programmed cell death 1
PD-L1: Programmed death-ligand 1
PFS: Progression-free survival
RCC: Renal cell carcinoma
TBP: Treatment beyond first progression
TCR: T-cell receptor
TIGIT: T-cell immunoglobulin and ITIM domain (TIGIT)
TILs: Tumor-infiltrating lymphocytes
TKI: Tyrosine kinase inhibitor
TME: Tumor microenvironment
TPS: Tumor proportion score
TTF: Time-to-treatment failure
UC: Urothelial carcinoma
VEGF: Vascular endothelial growth factor
